# Clinical and imaging diagnosis of pediatric testicular microlithiasis: a physician’s dilemma

**DOI:** 10.3389/fped.2025.1554081

**Published:** 2025-07-30

**Authors:** Ghada Habachi, Yosra Kerkeni, Jouini Riadh

**Affiliations:** ^1^ Faculty of Medicine, Tunis El Manar University, Tunis, Tunisia; ^2^ Department of Pediatric Surgery “A”, “Bechir Hamza” Children’s Hospital of Tunis, Tunis El Manar University, Tunis, Tunisia; ^3^ Research Laboratory LR12SP13, Faculty of Medicine of Monastir, Tunis, Tunisia

**Keywords:** testicular lithiasis, testicular calculi, children, testicular tumors, management

## Abstract

Testicular microlithiasis (TM) is a relatively rare and incompletely understood condition, particularly in the pediatric population. Its clinical significance and optimal diagnostic and therapeutic management remain subjects of ongoing debate. In order to clarify current practices and guide clinical decision-making, we conducted a literature review of recent studies published using the search terms *testicular microlithiasis*, *testicular calculi*, *testicular neoplasm*, and *children*. The primary objective of this review was to propose a standardized diagnostic management algorithm based on the available evidence. The nature of testicular microlithiasis remains a subject of ongoing debate. In the absence of definitive evidence, continued follow-up appears to be the safest approach to minimize the risk of delayed diagnosis in the event of malignant transformation or tumor development. Routine scrotal examination should be encouraged and properly taught, particularly to adolescents and their caregivers. Ultrasonographic (US) surveillance, while not mandatory, should be considered when accessible, especially in individuals with additional risk factors.

## 1 Introduction

Since the late 1900, TM has been described by Doherty et al (1) in a 10 year-old boy. US discovered tiny bright echoes scattered throughout an undescended testis. By the age of 23, the atrophic operated testis was removed and studied. Histology revealed calcific concretions in the seminiferous tubules and no tumors, thus the discovery of TM.

Since then, multiple case reports and series have reported TM in males from childhood to old age. In pediatric studies, its incidence varies from 1% to 6% in asymptomatic boys and is higher in a variety of associated conditions (2–5). In adult male population, it is comparable and varies from 2% to 5% (6). Before, the diagnosis was histological and established by biopsy or orchidectomy. However, nowadays, the diagnosis relies on scrotal US which could explain the increase of the reported incidence as well as the advances of the high-frequency US technology and the increased awareness of pediatric surgeons, urologists and radiologists. It is characterized by the presence of small ultrasonic foci scattered throughout the parenchyma.

Nonetheless, more than a decade later from its first identification, multiples questions remain unanswered with the absence of evidence-based guidelines in children. What causes TM in children? Is the lesion benign or premalignant and what is the natural course of evolution? What is the optimal management and follow-up regimen?

A comprehensive literature search was conducted using the PubMed and ScienceDirect databases. The search strategy included the following keywords: *testicular microlithiasis*, *testicular calculi*, *testicular neoplasm*, and *children*. Additional studies were identified by manually screening the reference lists of relevant review articles. No restrictions were applied regarding publication date or article type during the initial selection phase. However, only studies published in English or French were included. Titles and abstracts were screened for relevance, and eligible articles were subsequently reviewed in full.

The aim of this review is to develop guidelines from the literature in order to aid clinicians in the decision-making for these children.

## 2 Etiology

The etiology of TM remains undefined with multiple possible hypotheses. One hypothesis include the breakdown of tubular basement membrane and the liquefaction of a spermatocyte's dendrites causing accumulation of cellular debris, inflammation and calcification in the lumen of the seminiferous tubule (7, 8). Moreover, multiples authors have suggested the role of immune response and multifocal Sertoli cell dysfunction with insufficient phagocytosis of luminal degenerative cells (9). These theories could explain the frequency of TM in children as cellular turnover is more rapid in children causing more luminal debris.

Another theory postulates an abnormal calcic deposits in the seminiferous tubules (10) and Zhang et al (11) suggested the role of a nanobacterial infection in the genesis of the pathology. The exact etiology remains unclear and future studies are important as the understanding of the pathogenesis could help elucidate the presence or the absence of tumoral potential.

## 3 Clinical presentation

Usually TM is painless and impalpable with a fortuitous radiological diagnosis in asymptomatic patients with scrotal trauma, undescended testis or other. Some frequent associated conditions are testicular atrophy, testicular torsion, varicocele, hypogonadism and infertility.

The association with a patent processus vaginalis is frequent in patients with TM, such as hydrocele and cryptorchidism (12) though the causality remains unclear. Furthermore, Geode et al. have reported a lower incidence of TM in boys with undescended testis compared to normal boys, 2.8% vs. 4.2% respectively (2, 13) and Bach et al. has reported cases of TM in the contralateral normal testes in operated children (14). Therefore, the association is worth further studies. On the other hand, in operated patients, the prevalence is higher and could reach 20% in some series which could incriminate an operative trauma caused by vascular damage (13, 15).

Extratesticular calcifications could be associated in some genetic conditions such as the SLC34A2 gene mutation (4p15) that is associated with pulmonary alveolar microlithiasis (16). Central nervous system microlithiasis could also be associated in various conditions.

Multiple other genetic diseases have been reported with the most frequent association in Down's syndrome where overall prevalence could reach 22.8% of children (17). This could be due to the associated hypogonadism and atrophy in this population group (17). Yet in adults, TM is frequently associated with two genetic disorders, Down's syndrome and Kleinfelter's syndrome with an overall prevalence at 36% (18) and 17% (19), respectively. In children, the association with Kleinfelter's syndrome has rarely been reported. Moreover, there have been reports of TM in siblings of patients suggesting a genetic factor (20–22).

Although testicular microlithiasis (TM) is often an incidental and asymptomatic finding, some patients may present with scrotal symptoms, including pain, swelling, or increased testicular volume. And in certain series, pain was suggested as clinical manifestation of TM, with some reports indicating it as the leading reason for hospital admission among children diagnosed with TM in the absence of any other associated pathology (23). One hypothesized mechanism for TM-related pain involves the distension of the seminiferous tubules caused by intratubular calcifications.

TM is typically bilateral and diffusely distributed; however, cases of asymmetric or unilateral involvement have been documented (24). The pattern of microlith distribution can vary and may be focal, multifocal, or diffuse.

## 4 Diagnosis

### 4.1 Histological diagnosis

As aforementioned, TM was first identified via histology post orchidectomy. It is associated with a distinct histological appearance with a central dense calcium core surrounded by concentrically layered collagen fibers, organelles and vesicles that gradually deposit within the tubules (25). These characteristics are responsible for the radiological appearances as they present with multiple, non shadowing echoes measuring around 1–2 mm and randomly scattered throughout the testicular parenchyma (26). Nevertheless, hematoxylin bodies classifies by Rensahw as non lamellated calcifications, usually seen in germ cell tumors and burned out tumors, could have the same ultrasonic features and could only be differentiated via histological studies (26). Conversely, some calcifications are lost during tissue section and fixing in testicular biopsies (27).

### 4.2 Radiological diagnosis

**Figure 1:**
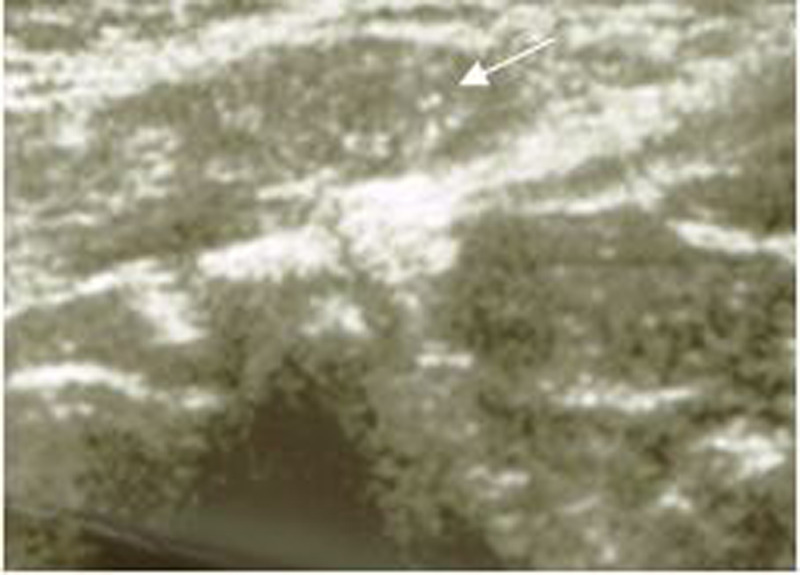
Testicular microlithiasis in a 6-year-old child. Longitudinal US of the right testicle shows microlithiasis (white arrow) with no tumor.

The diagnosis of TM relies on high-frequency US (7–10 MHz) as the testicles are ideal for superficial examination due to their extra-abdominal location producing high resolution images (28). TM are defined as small calcifications measuring from 1 to 3 mm in diameter, multiple, non-shadowing with no loss of testicular shape or volume (Figure 1) (24). The absence of posterior acoustic shadowing was explained by the small size of the foci (5).

US represent the Gold-standard especially in children as it is radiation-free, widely available, and low cost with no need for general anesthesia during the exam. The use of linear-array probes with high resolution may aid in detecting the smallest foci (29). Moreover, studies have reported good consistency and comparable inter-observer results (k = 0.86) (30). The low variability could be secondary to the clear radiological definition of TM and the standard classification.

Yee et al. (31) have graded the TM based on the number of microliths count in any single view. Limited TM is defined as less than five microliths per view and classic TM as more than five microliths per view. From 1 to 5 it has been graded as minimal/mild (grade I: 5–10 microliths), moderate (grade II: 10–20 microliths), and severe (grade III: >20 microliths). However, there was no significant difference in testicular tumor development between grades (31, 32).

In 2015, the European Society of Urogenital Radiology have proposed a new classification with 3 grades; limited from 1 to 4 per field of vision, classic from 5 and higher and diffuse or “snowstorm” (33). Similarly, these three grades have no significant difference regarding the tumor development.

Differential diagnosis of such echogenic foci include various conditions especially in case of limited TM. It varies from orchitis and arthritis to granulomas, scars and tumors (44). Testicular tumors are generally associated with larger and coarser calcifications (24). However diagnosis is usually made easier by the regularity and small-size of these foci (34) and in doubtful cases, MRI could be helpful.

TM are not detected on magnetic resonance imaging (MRI) on both T1- and T2-weighted images which makes diagnosing suspected testicular masses easier (35). Shear-wave elastography has been an interesting non invasive tool that evaluates vescoelastic tissue properties (36). It had demonstrated an increased elasticity in testes with microlithiasis compared to normal testes in children (37). However, the applicability of this technique is unclear.

## 5 Management of children with TM

The nature of TM remains a subject of ongoing debate, particularly regarding whether it represents a benign incidental finding or a premalignant condition, and whether spontaneous regression is possible. A systematic review identified testicular tumors in 15 children with TM, corresponding to an overall prevalence of 3.6% (38). In contrast, pediatric testicular tumors account for only 1%–2% of all solid tumors in children, suggesting a possible—but unconfirmed—association between TM and malignancy (39). Yet to date, no definitive evidence supports the classification of TM as a premalignant lesion. The majority of authors consider isolated TM to be a harmless finding and emphasize the importance of follow-up only in the presence of associated risk factors, such as undescended testis, testicular atrophy, a family history of testicular tumors, Klinefelter syndrome, hypospadias, or infertility.

The core of the debate lies in whether isolated TM warrants further investigation or should simply be regarded as a benign incidental radiological finding. Some authors consider further follow-up an overestimation of the clinical significance of TM and a source of anxiety for both children and their families, potentially impacting their quality of life and contributing to unnecessary strain on healthcare resources. As such, they advocate for no follow-up in asymptomatic patients without risk factors (40). Additionally, there have been reports suggesting the possibility of spontaneous regression of TM (12, 41).

Nevertheless, given the existence of case reports documenting tumor development in patients with TM, the potential risk cannot be entirely dismissed. Consequently, some experts recommend monthly testicular self-examination. Parents, guardians, and children should be properly educated on how to perform scrotal self-examination and made aware of the potential risks, in order to prevent diagnostic delays (40, 42, 43). Several cases have demonstrated the importance of self-examination in identifying palpable masses early, thereby enabling timely intervention (44). Whether annual clinical consultation and examination is required is still a matter of debate. Nonetheless, even if not mandatory, such visits provide a valuable opportunity to reinforce the importance of self-examination and encourage active patient engagement and awareness (45).

In addition, in several studies US has shown utility in detecting carcinoma *in situ* before lesions become clinically palpable (46), reinforcing its value as a follow-up tool (45, 47). However, its indication is primarily supported in patients with additional risk factors, such as testicular infertility (47, 48), testicular atrophy (49, 50), personal history (51) and cryptorchidism (52), where the risk of malignant transformation may be higher.

The European Society of Urogenital Radiology recommends annual scrotal ultrasonography until the age of 55 in patients with additional risk factors (33). Similarly, Goede et al. concluded that the risk of developing malignant testicular tumors is highest in boys older than 15 years (2). However, several reports have documented the occurrence of malignancy in younger children, highlighting the ongoing controversy and the need for vigilance in early adolescence and even childhood (3, 9, 53, 54). Given this potential for early malignant transformation, periodic scrotal examination by both parents and healthcare providers is advised, along with the introduction of self-examination practices in pubertal boys (44).

Testicular biopsy has no place in asymptomatic patients due to its invasive nature and associated risks (50). Its use should be limited to specific high-risk scenarios, such as evaluation of the contralateral testis in patients with known germ cell tumors (33, 55, 56) or in the context of significant testicular atrophy (33). In select cases, MRI may serve as a less invasive and effective alternative for further evaluation (56).

## 6 Actionable recommendations

In summary, monthly testicular self-examination seems the better cost-effective approach; though it is supported by low- to moderate-quality evidence, primarily derived from observational studies and expert consensus. Annual physical examination by a physician remains essential, not only for clinical assessment but also to reinforce patient education and compliance, and is supported by moderate-quality evidence.

Annual scrotal US may be considered for patients with additional risk factors and should be provided on patient request and if accessible. While the supporting evidence is of moderate quality, routine imaging could contribute to the early detection of non-palpable lesions and contribute to future efforts in establishing standardized, evidence-based guidelines.

Conversely, the use of tumor markers, CT or testicular biopsy is not recommended in asymptomatic individuals in the absence of clinical suspicion of malignancy.

A plan for transition to adult urology is mandatory due to the risk of malignancy and future infertility (8, 57, 58).

**Figure 2:**
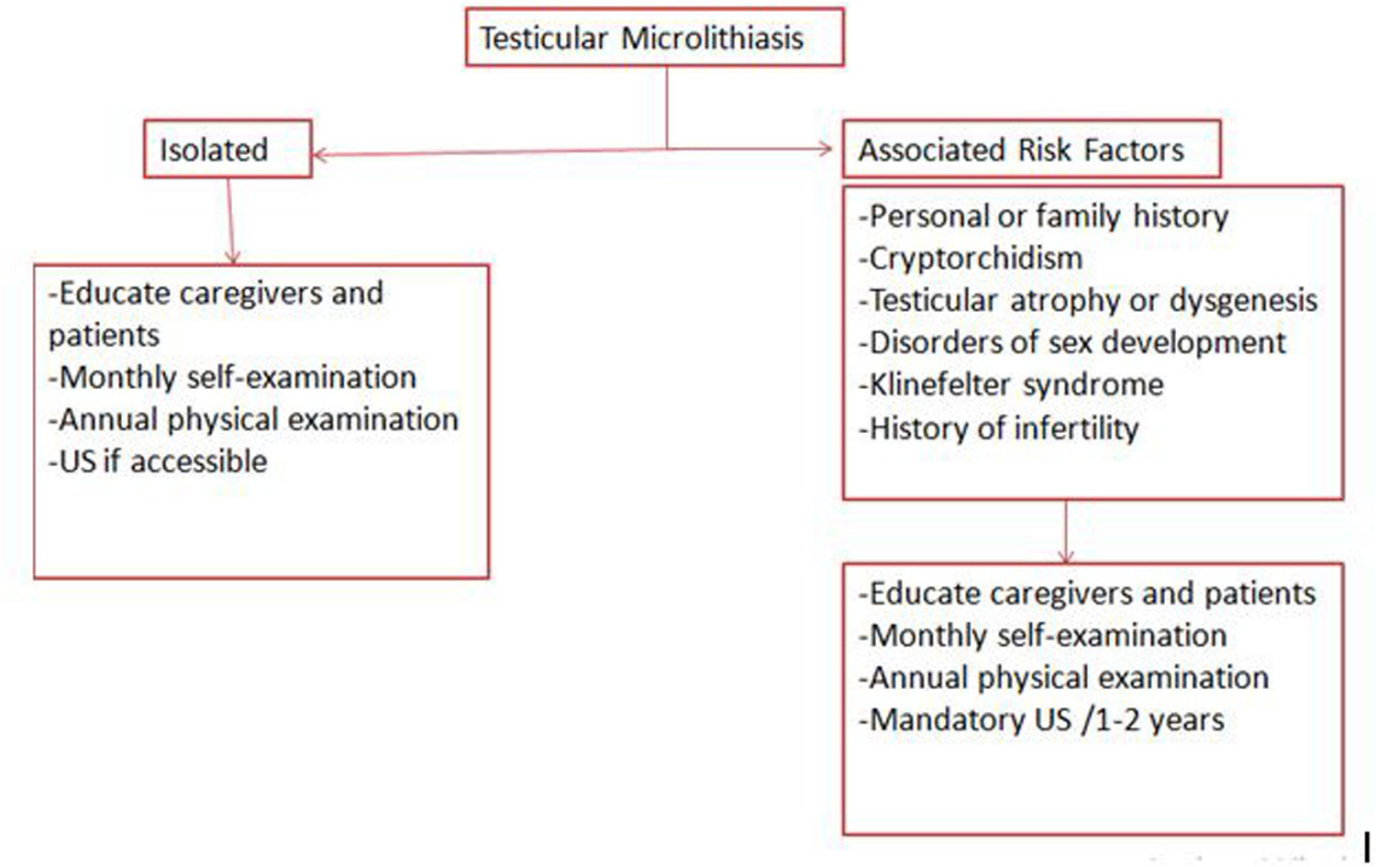
Proposed follow-up strategy for patients with testicular microlithiasis; recommendations are based on available clinical guidelines and literature: education and self-examination (low to moderate quality), annual physical examination (moderate quality), and periodic ultrasound surveillance in high-risk groups (moderate quality).

The proposed follow-up strategy is shown in Figure 2.

## 7 Discussion

More than a decade later, testicular microlithiasis (TM) remains a challenging radiological finding, with no established evidence-based guidelines for its management. This uncertainty largely stems from discrepancies in study populations, methodologies, and interpretations of risk. Management strategies vary widely depending on whether TM is regarded as a potential premalignant condition or merely an incidental and benign finding. Cases of malignant transformation have been reported in both pediatric and adult populations, supporting the rationale for long-term follow-up (6, 59). However, overly aggressive management approaches—particularly in young, asymptomatic patients—may lead to unnecessary anxiety, especially in the absence of robust evidence to justify such surveillance.

Clinical examination has traditionally been considered the gold standard in most series, having demonstrated its efficacy in the early detection of testicular abnormalities (44). However, recent guidelines from the European Society of Paediatric Radiology (61) and the European Association of Urology—Sexual and Reproductive Health advise against routine follow-up in men without associated risk factors, citing the lack of a consistent correlation between TM and testicular malignancy (62, 63). Nonetheless, this recommendation has not been validated in the pediatric population. In contrast, most pediatric studies continue to advocate for follow-up, even in cases of isolated TM, due to the limited data and potential risk of malignancy in this age group (33, 64).

In the presence of associated risk factors, there is general consensus on the importance of regular follow-up (43, 43). However, the role of imaging in these cases remains debated. Semi-annual, annual, or biennial scrotal ultrasound has traditionally been recommended due to its demonstrated efficacy in the early detection of non-palpable testicular lesions. Nevertheless, concerns regarding cost-effectiveness and patient burden have led some clinicians to adopt a more conservative approach, favoring clinical follow-up and self-examination alone (24). Nevertheless, the majority of authors continue to support the use of imaging—and, when indicated, biopsy—on a case-by-case basis (18, 33, 42, 52, 65). Chromosomal and biochemical investigations are generally reserved for individuals presenting with clinical features suggestive of an underlying chromosomal disorder (60).

We started this literature investigation in order to address questions arising from our clinical practice and to clarify ongoing controversies surrounding TM. The nature of the lesion remains a questionable matter, yet the need for appropriate follow-up is widely acknowledged. Various surveillance strategies have shown favorable outcomes, but the absence of high-level evidence continues to hinder the development of standardized recommendations. Given the potential risk of malignancy and infertility, the formulation of an optimal follow-up protocol remains a clinical challenge.

Patient education on testicular self-examination, along with annual clinical assessments, is essential to avoid delayed diagnosis of malignant lesions and unnecessary orchidectomy. While the cost of US may limit its routine use, we advocate for its annual implementation—particularly in patients with associated risk factors—when resources allow.

Ultimately, prospective pediatric studies are crucial to establishing robust, evidence-based guidelines for the management of TM.
